# Type A Aortic Dissection Following Heart Transplantation

**DOI:** 10.21470/1678-9741-2023-0252

**Published:** 2024-07-15

**Authors:** Alvaro Diego Peña, Eduardo Alberto Cadavid, Mayra Estacio, Alejandro Moreno-Angarita, Hector G Olaya R, Stephany Olaya

**Affiliations:** 1 Department of Cardiovascular Surgery, Fundación Valle del Lili, Cali, Valle del Cauca, Colombia.; 2 Deparment of Internal Medicine, Fundación Valle del Lili, Valle del Cauca, Colombia; 3 Centro de Investigaciones Clínicas, Fundación Valle del Lili, Cali, Valle del Cauca, Colombia; 4 Facultad de Ciencias de la Salud, Fundación Universitaria de Ciencias de la Salud, Bogotá, Colombia.

**Keywords:** Thoracic Aorta, Catheterization, Deep Hypothermia Induced Circulatory Arrest, Neuroprotection, Aortic Diseases, Thoracic Aorta Dissection

## Abstract

Cannulation strategies in aortic arch surgeries are a matter of immense discussion. Majority of time deep hypothermic circulatory arrest (DHCA) is the way out, but it does come with its set of demerits. Here we demonstrate a case with aortic arch dissection dealt with dual cannulation strategy in axillary and femoral artery without need for DHCA and ensuring complete neuroprotection of brain and spinal cord without hinderance of time factor. Inception of new ideas like this may decrease the need for DHCA and hence its drawbacks, thus decreasing the morbidity and mortality associated.

## INTRODUCTION

Aortic dissection is a rare but fatal complication following orthotopic heart transplantation. According to the underlying pathophysiology and the time of onset, patients can be categorized into four groups: the first category includes acute aortic rupture occurring in the early postoperative phase resulting from a mismatch between the donor and the recipient aorta; the second group comprises patients with postoperative infections resulting in mediastinitis and mycotic or bacterial pseudoaneurysms of the ascending aorta; the third group consists of patients with true aneurysms and dissection of the native aorta associated with a traditional cardiovascular risk factor; and aortic dissection in patients with heterotopic heart transplant makes up the final group^[1]^.

The diagnosis may be difficult because, in many cases, dissection may mimic various pathological states and could present without pain. It should be considered when dilatation of the ascending aorta or aortic arch and aortic valve regurgitation are present.

The appropriate surgical approach for patients with heart transplants and aortic dissection remains unknown. In this report, we describe the case of a type A aortic dissection in the native aorta that occurred 21 years after orthotopic heart transplantation and the surgical technique successfully applied for its repair.

## CASE PRESENTATION

A 52-year-old man with a pertinent history of antiphospholipid syndrome, deep venous thrombosis, and major depressive disorder underwent heart transplantation for end-stage ischemic cardiomyopathy in 2000. The patient was successfully discharged after heart transplantation.

**Fig. 1:**
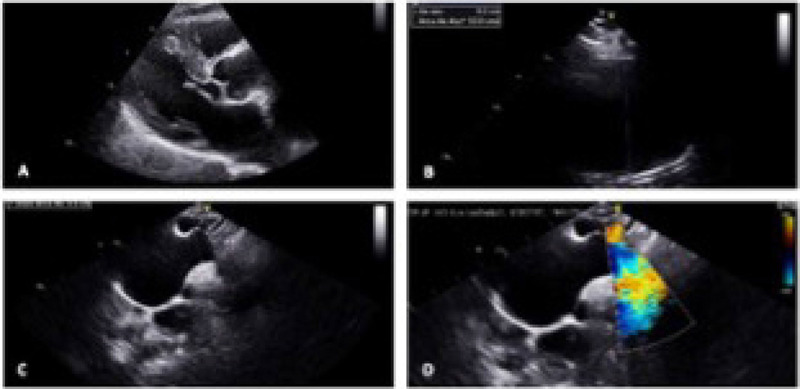
Transthoracic echocardiogram performed before surgery. A) Aortic valve and sinotubular junction. B) Ascending aorta aneurysm. C) and D) Distal portion of ascending aorta and Doppler color suggesting dissection.

In April 2021, during his routine examination, transthoracic echocardiogram (TTE) revealed a normal aortic valve and sinotubular junction with a large saccular ascending aortic aneurysm close to the aortic arch. Doppler color image acquisition suggested type A aortic dissection. Right and left ventricular functions were preserved (Figure 1).

**Fig. 2:**
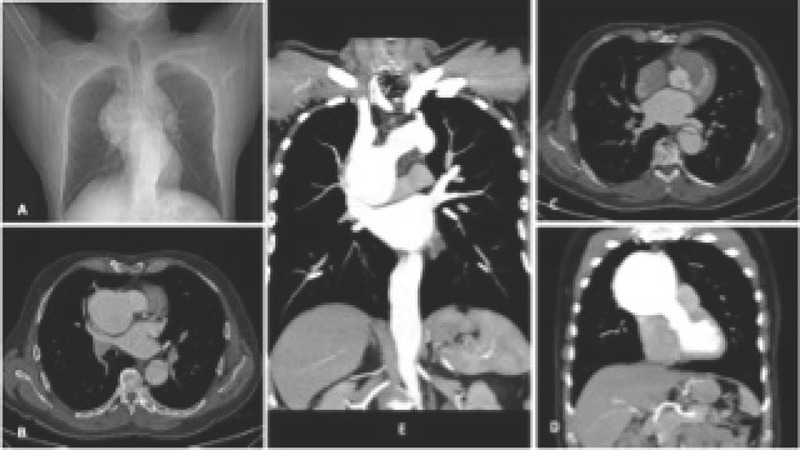
Thoracic aortic angiography. Dissection flap associated with native ascending aortic aneurysm with normal aortic valve ring. A) Relation of the aortic aneurysm with right lung. B) Origin of left main coronary artery and bifurcation. C) Right coronary artery origin. D) and E) Origin and end of the ascending aorta.

Computed tomography (CT) angiography of the chest confirmed the presence of a saccular ascending aortic aneurysm of 101 × 91 × 92 mm, associated with a dissection flap measuring 33 × 30 mm, originating above the sinotubular junction, involving the ascending aortic aneurysm, and extending to the iliac arteries. Both coronary arteries were well seen in a heart CT, showing the absence of obstructive disease or dissection (Figure 2).

### Surgical Treatment

**Fig. 3:**
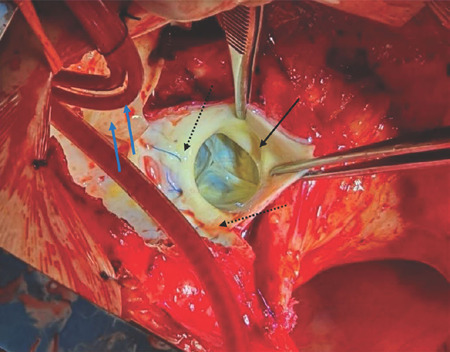
The anterior and sides of the aortic ascending aneurysm have been removed. Forceps are keeping together the aortic wall layers. Between them, right coronary artery ostium (black arrow) is visualized. Trileaflet aortic valve with excellent coaptation is observed, and the donor sinotubular junction is normal size. The posterior wall of the ascending aorta has not been dissected, and the recipient-donor aorta anastomosis suture line is shown (dash arrows). A circulatory arrest has been established at moderate hypothermia, and antegrade cerebral perfusion has been resumed by direct cannulation of the right and common carotid arteries (blue arrows).

The patient was taken to urgent surgery, and the operation was planned based on a TTE and CT angiogram of the aorta and heart. We first defined the location and length of the aortic dissection and aneurysm. From the images, we can see that the proximal part of the aorta, such as the aortic valve, coronary arteries, aortic root, and sinotubular junction corresponding to the donor side, was free from dissection and aneurysm. However, above it, there was a large aneurysm that involved the ascending aorta before the brachiocephalic trunk origin, and it was associated with a dissection flap that involved the aortic arch and descending aorta (Figure 3).

Peripheral cardiopulmonary bypass was instituted after percutaneous ultrasound-guided cannulation of the right femoral vein and artery. Transesophageal echocardiography was used to guide cannulation. In the arterial line, the guidewire was located in the true lumen, and in the vein line, the tip of the venous cannula was located 2 cm above the inferior vena cava. A medial sternotomy was performed intraoperatively, and many adhesions were observed in the ascending aorta that involved the pleura and lung parenchyma, corresponding to previously used hemostatic sealants. This made it difficult to release the aorta, so we decided not to separate the aorta’s back wall from it. The aorta was cross-clamped, the ascending aortic aneurysm was opened, and Custodiol® cardioplegia was given at 20 ml/kg by direct cannulation of the right and left coronary ostia.

**Fig. 4:**
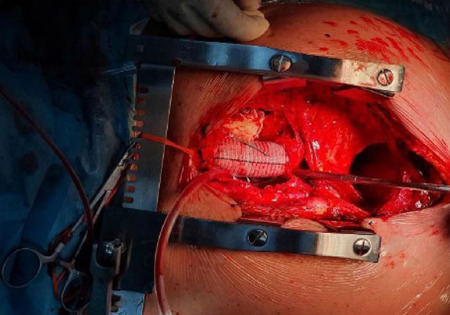
Replacement of the ascending aorta: proximal anastomosis is performed in the donor side aortic wall, and hemiarch (distal anastomosis) is completed with a 30 mm Dacron® graft tube and inclusion technique in the recipient aortic side (signaled by the arrows showing recipient aortic posterior wall remnants).

A large aneurysmal dilatation of the ascending aorta was then removed, and inspection of the aortic root showed normal morphology of the donor aortic valve, sinotubular junction, and coronary artery ostium (Figure 4). The aorta suture plane of the receptor-donor anastomosis was located within the aortic dissection. The ascending aorta was reconstructed with a 30 mm Dacron® Gelweave® synthetic graft. For the proximal donor-side anastomosis, we used a 3-0 polypropylene suture, placing deep stitches between the donor aortic wall (traveling 5 mm to 20 mm in depth in the posterior wall) and the Dacron® graft.

Once moderate hypothermia at 25 °C was reached, circulatory arrest and antegrade cerebral perfusion by direct cannulation of both right and common carotid arteries were given, and hemiarch or distal recipient side anastomosis was constructed. First, aortic wall layers were stuck back together by using separate stitches of pledged 3-0 polypropylene covering the circumference, and then native aorta Dacron® graft anastomosis was performed utilizing felp reinforced 3-0 polypropylene running suture. Circulatory arrest lasted 36 minutes.

Intraoperative echocardiographic verification showed good biventricular function and flow through the true aortic lumen. The aortic cross-clamping time was 55 minutes, and the cardiopulmonary bypass time was 108 minutes. Figure 4 showed postoperative imaging before sternal closure.

The operation was completed without complications. The patient was transferred to the intensive care unit and discharged 14 days after surgery. Postoperative therapy was without complications.

One year postoperatively, the patient remains in functional class I. A follow-up TTE showed trivial aortic insufficiency and normal biventricular function.

## DISCUSSION

Ascending aortic dissection is uncommon in heart transplant recipients. Its frequency after orthotopic heart transplantation varies between 1 and 2%, in a few cases reported in the literature^[1]^. This problem usually affects the native aorta, in patients with risk factors that are similar to those in the general population, like hypertension, Marfan syndrome, pre-existing aortic aneurysms, and atherosclerosis^[2,3]^.

Cardiac surgery other than retransplantation after heart transplantation is a challenge, and emergency procedures have high in-hospital mortality^[4]^. The first case of successful surgical treatment of aortic dissection confined to the donor aorta in a recipient of an orthotopic cardiac allograft was reported by Pak et al.^[2]^ in 1995. The appropriate management of transplant patients with Stanford type A dissection remains unknown. Most patients with acute aortic dissection are managed using open surgical techniques with a limited role for endovascular repair depending upon the extent of the dissection field^[5]^.

The strict graft inclusion technique for aortic root replacement represents a safe and feasible technique that avoids major complications like bleeding from injury to the coronary ostium, left atrial roof, and right pulmonary vein. In this case, we avoid any of those complications by limiting the surgical plane of dissection in the posterior aortic wall.

In our patient, we used hemiarch and ascending aorta replacement with the inclusion technique. With this method, the correction in the diameter of the ascending aorta corrects severe aortic insufficiency without touching the aortic valve since the main mechanism for this insufficiency is dilatation of the sinotubular junction and ascending aorta. The time for extracorporeal circulation was 108 minutes. Ali et al.^[6]^ reported an aortic cross-clamping time of 156 minutes with an extracorporeal circulation time of 222 minutes without the need for circulatory arrest. In published case reports, different surgical techniques have been described. In order to minimize the risk of infection resulting from immunosuppression, especially in the early period after transplantation, Teebken et al.^[7]^ performed an aortic replacement with human tissue to prevent the implantation of foreign material and optimize postoperative hemodynamics; however, they described a prolonged cross-clamping time.

In our institution, it is routine to close the pericardium in these patients, first to reduce the space left by the dilated native heart and thus reduce the accumulation of pericardial fluid and reduce the probability of inadvertent injury to the right ventricle and great vessels in a redo heart surgery; and second, to reduce adherence syndrome, which may even involve lung tissue in the great vessels and right atrium.

Another challenge of this entity is to recognize aortic dissection in transplant patients because it is often underdiagnosed. Dissection may mimic various pathological states, including myocardial infarction, heart failure, cardiogenic shock, and tamponade. As the heart is denervated, myocardial or aortic injury can also occur without pain, and mediastinal scarring may prevent or even delay aortic rupture^[1]^. As an option for timely screening and diagnosis, to perform CT of the heart annually in transplant patients could be chosen, which would allow not only the evaluation of coronary disease but also other complications such as aneurysms and aortic dissection. Fukuhara et al.^[8]^ have proposed annual follow-up of this diagnostic technique in patients with risk factors such as dilatation of the native aorta at the time of transplantation and refractory hypertension.

## CONCLUSION

We reported a successful strict graft inclusion technique for aortic root replacement to treat aortic dissection in a patient with history of heart transplantation 21 years before. It represents a safe and feasible method in patients in whom the aorta is firmly attached to the surrounding tissues and their separation could lead to irreparable vascular and large vessel injuries.

## Abbreviations, Acronyms & Symbols

CT: Computed tomography
TTE: Transthoracic echocardiogram
